# Roles of Abscisic Acid and Gibberellins in Stem/Root Tuber Development

**DOI:** 10.3390/ijms23094955

**Published:** 2022-04-29

**Authors:** Peilei Chen, Ruixue Yang, Dorothea Bartels, Tianyu Dong, Hongying Duan

**Affiliations:** 1 College of Life Sciences, Henan Normal University, Xinxiang 453007, China; chenpl521x@gmail.com (P.C.); 18737590152@163.com (R.Y.); dongty536@163.com (T.D.); 2 Institute of Molecular Physiology and Biotechnology of Plants (IMBIO), Faculty of Natural Sciences, University of Bonn, Kirschallee 1, D-53115 Bonn, Germany; dbartels@uni-bonn.de

**Keywords:** root and tuber crops, stem/root tuber development, GA, ABA

## Abstract

Root and tuber crops are of great importance. They not only contribute to feeding the population but also provide raw material for medicine and small-scale industries. The yield of the root and tuber crops is subject to the development of stem/root tubers, which involves the initiation, expansion, and maturation of storage organs. The formation of the storage organ is a highly intricate process, regulated by multiple phytohormones. Gibberellins (GAs) and abscisic acid (ABA), as antagonists, are essential regulators during stem/root tuber development. This review summarizes the current knowledge of the roles of GA and ABA during stem/root tuber development in various tuber crops.

## 1. Introduction

Root and tuber crops are characterized by their underground storage organs. These subterranean structures are rich in water, carbohydrates, and a range of proteins and secondary metabolites [1,2]. Root and tuber crops are indispensable for human food due to the storage contents, of which many are healthy or useful for various industrial applications [1,2,3,4]. Potato (*Solanum tuberosum*), sweet potato (*Ipomoea batatas*), and cassava (*Manihot esculenta*) are among the most important root and tuber crops. They not only feed billions of people, especially in the humid regions, but also are used as fodder because of the high levels of carbohydrates and proteins, as well as minerals and vitamins [1,3,4,5]. Yams (*Dioscorea* spp.) are the fourth-most-important tuber crop, which are viewed as both famine food during the food scarcity periods and medicines with pharmacological effects [6]. Other root and tuber crops, such as Chinese arrowhead (*Sagittaria trifolia*) [7], Chinese foxglove *(Rehmannia glutinosa)* [2], yacon (*Smallanthus sonchifolius*) [8], and sugar beet (*Beta vulgaris* L.) [9], also provide vegetables, fruits, medicine, industry materials, and economical values.

Plant root system architecture shows high diversity. There are various kinds of modified underground stems/roots among the root and tuber crops. Potato and yam tubers are belowground modified stems originating from horizontally growing stolons and vertical hypocotyls, respectively [10,11,12]. The tuberous roots of sweet potato and cassava develop from adventitious roots [1]. The morphological diversity of some other root and tuber crops are summarized in Table 1. In general, the edible organs of the root and tuber crops arise from the underground swollen stems or roots. These stem/root tubers usually go through three development stages: initiation, expansion/enlargement, and maturation [2,12,13] (or partitioned into induction, initiation, and tuberization in some literatures [14,15]). Potato tuber initiation starts with the expansion of the central pith tissue in the hooked stolon [1,4]. Subsequently, the enlargement of the perimedullary region, comprised of xylem, phloem, parenchyma, and meristematic cells, accelerates the tuber expansion and finally leads to the maturation of stem tuber [1]. The growth of tuberous roots in cassava is a result of the enlargement and division of cells in the vascular cylinder, which eventually develops into a storage zone mainly containing the xylem vessels and starch-storing xylem parenchyma cells [1]. The mature tubers will reach the final size and accumulate maximum nutrients, along with the decay of the overground leaf canopy and the dormancy of the underground tuber [2,12]. All physiological and morphological developments of stem/root tuber crops are regulated by a series of environment factors [15,16]. Potato tuber organogenesis is initiated by a short photoperiod and negatively regulated by other environment perturbations, for example, high night temperatures or high nitrogen levels [16]. Chinese yam tuberization is also initiated by short-day photoperiods, but further enlargement is suppressed under short-day photoperiods after rapid growth [12]. The effects of the environmental cues on tuber development require the finely tuned modulation of endogenous phytohormones [15,16]. Gibberellins (GAs) and abscisic acid (ABA) are two important phytohormones that are involved in various developmental processes, including tuberization with antagonistic [17,18,19]. In this review, we summarize the recent advances in understanding the functions of GA and ABA in the stem/root tuber development of different tuber crops.

## 2. Gibberellins in the Development of Stem/Root Tubers

Gibberellin (GA) is an umbrella phytohormone term encompassing a large family of tetracyclic diterpenoid carboxylic acids with the *ent*-gibberellane or 20-nor-*ent*-gibberellane carbon skeletons [20]. To date, more than 130 GAs have been identified [21]. However, the major bioactive GAs are restricted to GA_1_, GA_3_, GA_4_, and GA_7_, while others are considered as the precursors or the deactivated forms of bioactive GAs [21]. GA has been implicated in multiple developmental processes via dominant reinforcement of cell elongation or sometimes cell division, as exemplified by tuber growth [1,17,18,20,21,22,23]. Potato tuber formation is retarded because of the stolon elongation promoted by the exogeneous bioactive GA_4/7_ [18], while the yam tuber and bulbil yield are increased by the application of GA [23,24]. The opposite effects of GA treatments suggest the intricate roles of GA during tuber development in different stem/tuber root crops [23].

### 2.1. Gibberellin Biosynthesis and Deactivation

**Figure 1:**
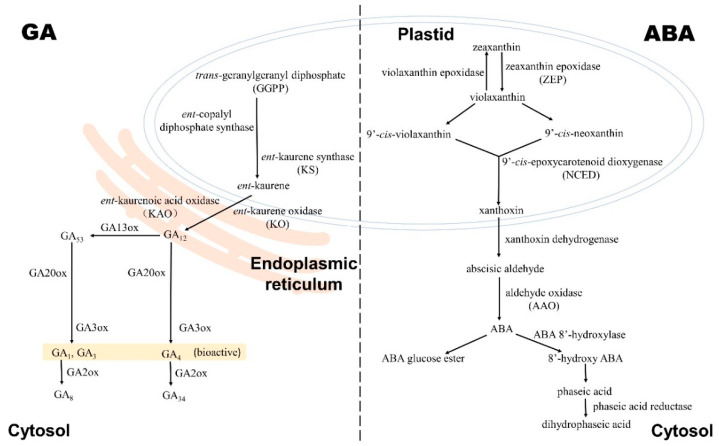
The major GA and ABA metabolism in tuber and root crops. The major GA and ABA metabolism (biosynthesis and catabolism) is predicated according to the expression patterns of genes for the key enzymes during stem/root tuber development and the detection of GA or ABA metabolites in stem/root tubers.

In plants, the common GA precursor is the biologically inactive GA_12_ [20,22,25]. It is transported through the plant vascular system as a mobile GA signal involved in plant developmental transitions [26]. The production of GA_12_ starts from *trans*-geranylgeranyl diphosphate (GGPP) in the proplastide [20,22,25]. GGPP is converted into the hydrocarbon intermediate *ent*-kaurene after catalyzation by *ent*-copalyl diphosphate synthase and *ent*-kaurene synthase (KS) [20,22,25] (Figure 1). In cassava, the transcript of the *KS* homolog was mainly detected in the cortex and parenchyma of fibrous roots and significantly downregulated as the storage root enlarged [27]. The conversion from *ent*-kaurene to GA_12_ occurs in the endoplasmic reticulum [20,22,25], which involves oxidation steps catalyzed by two membrane-associated cytochrome P450 mono-oxygenases: *ent*-kaurene oxidase (KO, localized in both plastid and endoplasmic reticulum), and *ent*-kaurenoic acid oxidase (KAO, localized in the endoplasmic reticulum) [20,22,25] (Figure 1). During corm formation in *S. trifolia*, the expression of *KS* and *KO* was enhanced in the process of stolon elongation and decreased when the corms swelled [28]. Both KS and KO are the key enzymes in the early steps of GA biosynthesis. Thus, the modification of *KS* and *KO* transcript expression indicated the reduced level of GA after tuber initiation. The observation is parallel to the report that the accumulated GA_1_ during potato tuber initiation was decreased conspicuously in the enlarged potato tuber [18]. The biosynthesis of bioactive GAs follows the divergence of GA_12_ into two branches in the cytosol, one leading to the formation of GA_4_ and the other to that of GA_1_ and GA_3_ [20,22,25]. GA_4_ is produced through the successive oxidization of GA_12_ by two soluble oxoglutarate-dependent dioxygenases (ODDs), GA20ox and GA3ox [20,22,25]. The formation of GA_1_ and GA_3_ goes through similar pathways after the transformation of GA_12_ to GA_53_ catalyzed by 13-hydroxylases (GA13oxs) [20,22,25] (Figure 1). The potato GA 20-oxidase gene, *StGA20ox1*, is expressed mainly in leaves, but it still intervenes in potato tuberization via the biosynthesis of bioactive GAs [29,30]. Under short-day photoperiods, the potato plants over-expressing *StGA20ox1* turned out to be taller, with longer internodes and delayed time of tuberization [30]. The opposite phenotypes, shorter stems, shorter internodes, and earlier tuberization, were observed in plants expressing antisense copies [30]. Another potato ODD gene, *StGA3ox*, has partial analogy with *StGA20ox1* [31,32]. The silencing of *StGA3ox1* using RNAi also resulted in smaller plants with shorter internodes, but the time of tuber initiation and the tuber yield were not affected despite induction of more tubers [31]. The over-expression of *StGA3ox1* in tubers also gave rise to slightly delayed tuberization, whereas earlier tuberization and taller plants were observed as *StGA3ox1* was over-expressed in leaves [32]. In the tuberous root of *R. glutinosa*, the diminution of *GA20ox* and *GA3ox* expression was consistent with the decline in the GA_3_ levels during root development [33]. These results concerning the two ODD genes demonstrate the negative effects of GA biosynthesis on tuber growth. GA may be involved in triggering similar downstream actions on sink tissue development regardless of tubers or tuberous roots originating from different organs [33].

GA is an important phytohormone implicated in multiple biological processes so that the concentration of the bioactive GAs is finely tuned. The tight regulation depends on both the synthesis and deactivation of bioactive GAs [22,25]. The classical bioactive deactivation was catalyzed by two major groups of ODDs. GA 2-oxidases (GA2oxs) are capable of converting the bioactive GA_1_ and GA_4_ into inactive GA_8_ and GA_34_, respectively, and transform some other GA intermediates [22,25] (Figure 1). In potato, the upregulation of the *StGA2ox1* transcript level was detected in the subapical zone of stolon and growing tubers before tuber expansion [34]. The reduction and over-expression of *StGA2ox1* in transformants caused late and earlier tuberization, respectively, by modifying the GA levels [34]. The phenotypes of the transformants showed that the implication of GA2oxs in GA metabolism exerts influence on tuber development as well. The regulation of *ODD* expression involving GA biosynthesis and deactivation has also been identified in turnip, carrot, and yam [23,35,36] (Table 1). Similar to potato, GA plays a negative role in taproot development as well because the swelling of the taproot in turnip or carrot is inhibited by exogenous GA [35,37,38]. The early development of taproot is associated with a decline in the content of endogenous active GA (GA_1_, GA_3_, GA_4_) and GA metabolites (GA_53_, GA_12_, and so on) [35]. Liu et al. ascribed the decrease in the GA content in turnip to GA biosynthesis but not GA bioactivation in the light of the downregulation of both biosynthesis ODDs (*BrrGA20ox*, *BrrGA3ox*, and *BrrGA13ox*) and the bioactivation ODD (most *BrrGA2ox* except for *BrrGA2ox8-4*) [35]. A similar expression trend of *ODD*s was detected in carrot [36]. The function of GA in the development of yam tubers is different to that in potato. The yam tuber yield was increased by treatment with GA, and the GA homeostasis in yam after treatment with GA_3_ was maintained as a result of the downregulation of *DoGA20ox1* and *DoGA3ox1* and the upregulation of *DoGA2ox3* and *DoGA2ox4* [23]. Besides the catalyzation of ODDs, there are two other GA deactivation mechanisms reported [22,25]. One rests with a cytochrome P450 monooxygenase, named EUI in rice, epoxidizing the 16,17-double bond of non13-hydroxylated GAs, and another one depends on the methylation of the C-6 carboxyl group of GAs with gibberellin methyltransferases [22,25]. So far, these genes are still not identified in stem/root tuber crops.

### 2.2. Gibberellin Signaling Pathway

**Figure 2:**
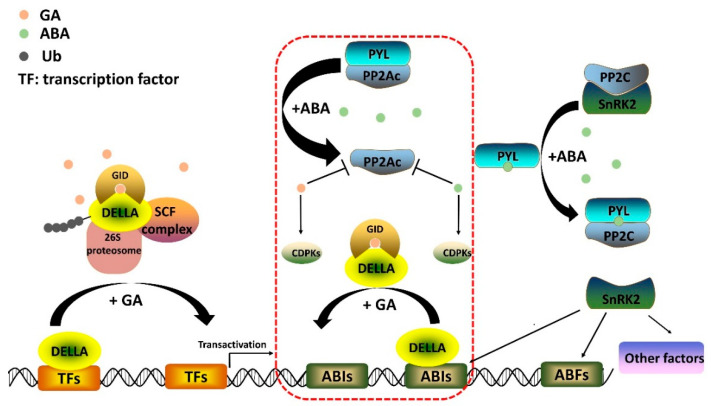
The potential GA and ABA signaling during stem/root tuber development. Arrows and linkers represent positive and negative effects, respectively. The components encircled by a red dotted rectangle line indicate the potential protein factors in both core GA and ABA signaling.

It is acknowledged that the GA signaling pathway (Figure 2) centers on DELLA proteins, which repress GA-dependent biological processes [39,40]. As the hub of GA signaling, DELLAs behave in a manner similar to the lock for the GA responsive genes, while GA acts as the pivotal part of the assembled key. The key can trigger the degradation of “the lock” DELLA and the transcriptional activation of GA responsive genes. Another key component of the unassembled key is the soluble nuclear GA receptor gibberellin-insensitive dwarf 1 (GID1) [39,40]. GID1 has the potential to bind GA molecules with a GA-binding pocket [39]. The binding between GID1 and bioactive GAs leads to a conformational change, and thus the assembled molecule becomes capable of turning on the expression of GA-responsive genes “locked” by DELLAs through forming the GA–GID1–DELLA complex [39,40]. Subsequently, the degradation of DELLAs is triggered by the SCF (SKP1, CULLIN, F-BOX) E3 ubiquitin–ligase complexes [39,40]. In rice and Arabidopsis, the F-box protein components of SCF were identified as GID2 and SLEEPY1 (SLY1), respectively, presiding over labeling DELLA using polyubiquitin chains for subsequent degradation by the 26S proteasome [39,40]. Ultimately, the GA downstream signaling cascade is stimulated along with the decrease in DELLAs [39,40]. This describes the upstream of the DELLA-dependent GA signaling pathway. However, DELLAs can exert negative impacts on GA signaling without degradation [39]. In the absence of SCF^SLY1/GID2^ activity, DELLAs also serve as transactivation factors whose transcriptional activity is inhibited by the GA–GID1 complex [39]. DELLAs are highly conserved [39]. DELLAs also have a major role in GA-induced development in stem/root tuber crops. The GA–GID1–DELLA regulatory module was identified in yam [23]. The interaction between DoGID1s and DoDELLAs in yeast was in a GA-mediated manner [23]. Moreover, the expression patterns of *DoGID1*s and *DoDELLA*s during tuber development and their responses to exogenous GA and the GA biosynthesis inhibitor paclobutrazol further substantiated the involvement of DELLA-dependent GA signaling pathways in yam tuber growth [23]. Tomato/potato heterografting (potato as rootstocks) caused a decreased potato tuber number [41]. The up-regulated expression of GA networking regulatory genes, *StDELLA* and *StGID1*, in rootstock was a hint for the conclusion that changes in DELLA-dependent GA signaling contribute to potato phenotypic modification [41]. DELLA-family proteins are versatile [39]. Their various negative regulatory functions are consistent with the roles of their target genes [39,40]. DELLAs are involved in flowering time regulation because of their interaction with *FLOWERING LOCUS T* activators, such as CONSTANS and PHYTOCHROME INTERACTING FACTOR4, and *FLOWERING LOCUS T* suppressors [17,39]. The expression of these genes was significantly regulated during both rhizome development and early flowering in lotus [42]. Cao et al. transferred *CONSTANS-LIKE 5* of lotus (*Nelumbo nucifera*) into potato and therefore achieved increased weights of potato tubers and starch content [43]. These results suggest that DELLA-mediated flowering signaling might regulate tuber growth as well. Except for the flowering regulation factors, other transcriptional factors associated with DELLAs were detected in stem/root tuber crops. In turnip, the interactions between DELLAs and NAC transcription factors, related to lignification of the secondary cell wall, resulted in GA-induced xylem lignification and thus the inhibition of taproot formation [35]. In Arabidopsis, the GA-biosynthetic enzyme GA 20-oxidase is also one of DELLAs’ target genes [44]. Therefore, DELLAs are major components of GA feedback regulation through interacting with another transcription factor GAF1 [44]. The GA feedback regulation of *GA20ox*s also exists in potato [45], but the involvement of DELLAs still needs to be verified.

Except for the DELLA-dependent GA signaling pathway, some studies have indicated alternative GA signaling without hinging on DELLA [46,47]. One of the putative DELLA-independent GA signals is involved in the mediation of cytosolic Ca^2+^ ([Ca^2+^] _cyt_) [46,47]. GA application can trigger an increase in the second messenger Ca^2+^ in the cytoplasm, even for *della* pentuple mutant plants [46,48]. The DELLA-independent increase in [Ca^2+^] _cyt_ may be induced through GID1 or some other membrane-localized GA receptor [46]. Ca^2+^-dependent protein kinases (CDPKs) are an important group of Ser/Thr protein kinases conveying signals to physiological responses. The expression of CDPKs was detected in tuberizing potato stolons and upregulated after the application of exogenous GA_3_ [49,50]. In cassava, the KS was viewed as involving the induction of CDPK expression according to similar expression patterns in the storage root initiation and early developmental stages [27]. In addition to CDPKs, another calcium-dependent protein, annexin, belonging to calcium-dependent phospholipid-binding proteins, was upregulated as a consequence of treatment with exogenous GA_3_ in potato [49]. The expression of the calcium-dependent proteins by the application of GA in potato suggests the GA-induced calcium-dependent signaling impacts on the inhibition of tuberization [49].

Both GA metabolism and signal transduction can affect plant development, spanning the entire plant life. The effects of GA metabolism depend on the regulation of the bioactive GA content level, which then further regulates the expression of target genes through DELLA-dependent and DELLA-independent signaling pathways. Since the last century, ample evidence exists from stem/root tuber crop studies that gibberellins are a negative regulator of tuber formation (Table 1). The inhibitory role of GA in tuber formation correlates with facilitating cell elongation. Over-expression of GA biosynthetic or metabolic enzymes tends to result in taller mutants with delayed tuberization (*GA20ox* and *GA3ox*) or dwarf plants with earlier tuberization (*GA2ox*) [30,31,32,34,40]. Moreover, the stimulation of root xylem development and lignification by GA is another reason for the inhibition of tuber expansion [35,37,38,51], as normally the downregulation of lignin biosynthesis genes and continuous decreasing lignin content take place at an early stage of tuber formation [52,53]. Nevertheless, GA is essential at some stages of tuberization. The cumulative bioactive GA in yam may induce cell expansion during early tuberization [23,34]. Cell enlargement during the later developmental stages of sugar beet taproot correlates with increased gibberellin levels resulting from the preferential expression of *GA20ox* and *GA3ox* during late development stages [54,55,56].

## 3. Abscisic Acid in the Development of Stem/Root Tubers

Abscisic acid (ABA) is an important sesquiterpenoid phytohormone. It is commonly considered as a stress hormone because of its crucial role in regulating adaptive responses, such as suberin biosynthesis, stomatal movement, and osmotic modification, in response to various stresses. However, basal ABA plays a significant role during plant growth and development as well, such as in bud and tuber dormancy and seed germination [57,58,59]. About twofold downregulation of ABA signaling pathway genes under drought stress in potato tubers indicates the important role of ABA in normal tuber development [60]. ABA was first found as a growth-inhibiting substance from peels of resting potatoes [57]. Afterward, retarded tuberization was observed in ABA-deficient potatoes [61]. A decreased endogenous ABA level was identified during potato stolon and tuber development, which counteracted GA and assisted in tuber development [18]. Stringent regulation of endogenous ABA levels involving ABA metabolism and signaling pathways has been well characterized, but how it exerts actions on tuber development is still not clear. Hitherto, there has been a paucity of studies focusing on the function of ABA in tuberization besides some omics analysis concerning tuber development. In this part, ABA metabolism and signaling implicated in tuber development will be summarized and discussed.

### 3.1. ABA Biosynthesis, Catabolism, and Conjunction

ABA belongs to terpenoid metabolites and contains 15 carbon atoms [57,62], yet its biosynthesis requires the cleavage of C_40_ carotenoid (indirect pathway) but not the C_15_ sesquiterpene precursor (direct pathway) in higher plants [57,62]. The first C_40_ precursor is the oxygenated C_40_ carotenoids, called zeaxanthin [63]. The conversion from zeaxanthin to the first C_15_ precursor xanthoxin in the de novo biosynthesis pathway occurs in plastids, involving several enzymes [57,62,63,64,65] (Figure 1). Zeaxanthin epoxidase (ZEP) catalyzes the conversion from zeaxanthin to violaxanthin by introducing oxygen into zeaxanthin [63]. This reaction can be reversed by violaxanthin epoxidase [63]. Violaxanthin is then isomerized into 9′-*cis*-violaxanthin by an unknown isomerase or 9′-*cis*-neoxanthin with the catalyzation of neoxanthin synthase and some isomerase [57,62,63,64,65]. Subsequently, the key rate-limiting enzyme NCEDs (9′-*cis*-epoxycarotenoid dioxygenases) oxidatively cleaves the two *cis*-xanthophylls into the C_15_ precursor xanthoxin [57,62,63,64,65]. In sugar beet, *ZEP* was preferentially expressed during the late development of beet according to the transcript profiles [55]. Similarly, the expression of *ZEP*s and *NECD*s was elevated at the final stages of yam aerial tuber (bulbils) development as well. The expression pattern conformed to the endogenous ABA content change in bulbil tissue [24]. The genes encoding key ABA biosynthetic enzymes StZEP and StNCED have been identified in potato tubers, but only their roles in tuber dormancy have been meticulously analyzed [66,67]. Römer et al. succeeded in improving the carotenoid content level in potato tuber by inhibiting the synthesis of violaxanthin in transformants using the sense and antisense constructs encoding *ZEP* [68]. However, no growth or tuber yield difference was observed between wild-type and transgenic plants [68]. This result may be attributed to the unchanged ABA content in transformants, which suggests the possibility of ZEP isoforms and redundancy of ABA biosynthesis pathways. Except for potato, the *ZEP* gene was also cloned and characterized in *Pseudostellaria heterophylla* [69]. It was upregulated along with the development of tuberous roots after flowering and responded to fluridone (the inhibitor of ABA biosynthesis) [69]. ABA biosynthesis moves to the cytosol accompanied by the transportation of the C_15_ precursor xanthoxin. In cytosol, xanthoxin is converted into abscisic aldehyde by xanthoxin dehydrogenase and finally oxidized to ABA by aldehyde oxidase (AAO) (Figure 1). In *R. glutinosa*, the increased endogenous ABA level during tuberous root development was correlated with the expression of ABA biosynthesis genes encoding ZEP, NCED, and AAO [33]. The similar overall enhanced expression of ABA biosynthesis genes was also detected in the development of carrot roots [36]. However, in sweet potato the expression of the *AAO* gene was enhanced at the early stage of storage root development but then sharply decreased until the final stage of storage root development [70].

The modulation of active ABA homoeostasis is reminiscent of GA, requiring not only biosynthesis but catabolism and conjunction as well [57,62,63,64,65] (Figure 1). Irreversible catabolism is the hydroxylation of ABA at three different methyl groups: C-7′, C-8′ (primary catabolic route), and C-9′ [57,63]. Among the three hydroxylation products, 8′- and 9′-hydroxy ABA, generated by the catalyzation of the CYP707A subfamily of P450 monooxygenases, are then isomerized spontaneously into phaseic acid and neophaseic acid respectively [63]. Phaseic acid with partial biological activity is finally converted into the inactive dihydrophaseic acid by phaseic acid reductase [63]. In line with the expression of the ABA biosynthesis genes in *R. glutinosa* and carrot, the ABA catabolism genes encoding ABA 8′-hydroxylase were concomitantly downregulated [33,36]. The expression patterns carried a potential for triggering the elevation of endogenous ABA levels during storage organ development [33,36]. The expression of *ABA 8-hydroxylase* in *S. trifolia* was also decreased during corm formation, but the downregulation only took place at the initial swelling stage but not during later stages of stolon development [28]. The modulated expression of ABA biosynthesis and catabolism genes in various species indicates the potential function of ABA in stem/root tuber development. Another pathway involved in the dynamic equilibrium of ABA is the reversible conjugation of ABA with glucose. This pathway generates inactive ABA glucosyl esters by the catalyzing addition of glucose moieties via uridine diphosphate glucosyl transferases [62,63]. The product can sequester ABA and thus serves as a transport form or as a storage reservoir [62,63]. Although the intermediate products, dihydrophaseic acid, phaseic acid, and ABA glucose ester, have been detected in root/stem tubers [66,71], genes involving ABA catabolism and conjugation, except for *ABA 8-hydroxylase*, have not been identified in the development of stem/root tubers yet.

### 3.2. Core ABA Signaling Pathway

The core components of the ABA signaling pathway encompass the intracellular receptor, pyrabactin resistance-like proteins (PYLs), the co-receptor, clade A protein phosphatases of type 2Cs (PP2Cs), and sucrose non-fermenting-1 (SNF1)-related protein kinase 2s (SnRK2s) [57,58,63,64,65] (Figure 2). The soluble receptor PYLs are localized in the cytosol and nucleus [64]. The direct binding of PYLs to ABA elicits conformational changes in PYLs, which gives rise to the interface in PLYs for interacting with PP2Cs [64]. The expression of group A PP2Cs can be intensively induced by the application of exogenous ABA [72]. The increased endogenous ABA level and its concomitant upregulation of PP2Cs were observed in the early microtuber formation of *Dioscorea opposite* [73]. The accumulation of transcripts of PLYs and PP2Cs was also detected during radish taproot thickening and *R. glutinosa* tuberous root formation [74,75]. The interaction between PYLs and PP2Cs releases SnRK2s due to the occupation of the enzymatic active site of PP2Cs, and then SnRK2s are activated by autophosphorylation [57,58,64]. SnRK2s are the positive regulators in ABA signaling. Many downstream components, including membrane channels, transcription factors, and transporters, are controlled by SnRK2s [57]. When SnRK2s are released from PP2Cs, the activity of ABA-responsive element binding proteins/factors (ABEBs/ABFs) and ABA INSENSITIVE 5 (ABI5), belonging to the basic leucine zipper domain (bZIP) transcription factors, is stimulated and thereby the ABA-responsive gene transcription is actuated [76,77]. The expression of yam *ABF3* at the final stage of bulbil growth was three times higher than during early stages, suggesting enhanced ABA signaling at later stages of yam bulbil growth [24]. The constitutive expression of *AtABF4* in potato not only led to the increased number of tubers per plant and improved tuber yield under non-stress conditions but also induced enhanced salt and drought tolerance [78]. However, this result could not be repeated using another bZIP transcription factor of hot pepper (*Capsicum annuum*) [79]. The heterologous expression of *CaBZ1* in potato only improved the tuber drought tolerance, while no phenotypic change was observed under non-stress conditions [79]. The different results suggest that not all bZIP transcription factors involving ABA signaling are associated with tuber development. Transcriptomic analyses of the rhizome formation in lotus and taproot thickening in *Panax notoginseng* and carrot show that numerous ABA core signaling component genes are regulated differently during tuber development [36,42,80] (Table 1). Nonetheless, the genes related to ABA signaling in some species exhibited a consistent expression trend, i.e., significant upregulation in the early and late root development stages in *P. notoginseng* and downregulation in carrot roots after 40 days of growing [36,80]. The different expression patterns in different species present the different ABA signaling pathways in modulating tuber development in various species.

It is obvious from the forementioned studies that the ABA metabolism and signaling pathway participates in stem/root tuber formation. ABA plays a positive role in the process of tuber development. In sweet potato, much more endogenous ABA was determined in the storage roots compared to that in the non-storage roots and in the heavier tuberous roots of cultivars compared to that in the lighter tuberous roots of wild species [70,81]. Tuberous root development displayed a positive correlation with ABA contents. The facilitated yam microtuber formation by the application of ABA and the significantly arrested microtuber development by ABA inhibitors further confirm the positive function of ABA [73]. In addition, ABA content increases along with the development of yam bulbs [24]. These results further suggest that ABA plays a major role in the maturation and storability of bulbs or tubers. The positive influence of ABA on tuber development may be associated with its role in sugar metabolism [33], as ABA implies a potential for stimulating transport of sugars derived from source tissues to sink tissues, for instance, the storage stem/root tubers [82].

## 4. Conclusions: The Antagonistic Role of GA and ABA in the Development of Stem/Root Tubers

**Figure 3:**
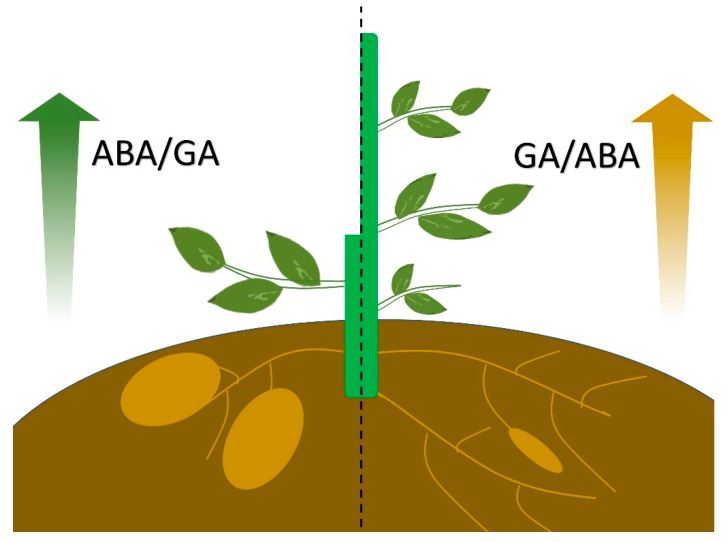
A model showing the antagonistic role of GA and ABA during tuber development. A higher ABA/GA ratio leads to the tuberization, while a higher GA/ABA ratio results in longer stems and delayed tuberization.

GA and ABA play antagonistic roles during tuber formation. GA represses tuber swelling, while ABA stimulates tuber formation (Figure 3). Furthermore, the death of tubers in an ABA-deficient droopy potato mutant was stopped by the application of the GA inhibitor tetcyclacis [18,61]. The observation demonstrates that ABA exerts positive effects on tuberization by counteracting the negative effect of GA [18,61]. The profiles of endogenous ABA and GA levels during the process of tuberization in various species are different (Table 1). In potato, the endogenous GA_1_ reached a peak during stolon elongation when the ABA level decreased and then GA_1_ decreased during tuber swelling when a little more ABA was accumulated [18]. The ABA level in *R. glutinosa* exhibited a continuous increase during tuberous root development, yet GA was reduced after 45 days of growing [33]. However, in sweet potato, continuous increase was detected for GA, and a strikingly increased ABA level was observed at the early stage, which then decreased along with storage root development, with a minor rise in the late development stage [70]. The diverse species-specific profiles of ABA and GA content during tuber formation imply the complexity and diversity of the mechanisms underlying stem/root tuber development.

The antagonistic regulatory roles of GA and ABA are implicated in various stages of plant development, i.e., during tuber formation and also during seed maturation and dormancy, root growth, and flowering [19]. Numerous factors are relevant to the ABA and GA antagonism function in diverse biological processes. In the model plant *Arabidopsis thaliana*, during post-embryonic root development, a C2H2-type zinc finger, GA-AND ABA-RESPONSIVE ZINC FINGER, is regulated by both GA and ABA, which in turn intervenes in the transcriptional regulation of ABA and GA homeostasis [83]. A C3H-type zinc finger SOMNUS, associated with seed germination, is controlled by the two phytohormones as well, whose promoter is targeted by both GA and ABA signaling components, DELLAs and ABIs [84]. Liu et al. summarized the antagonistic interaction between core GA- and ABA-signaling components [85]. However, the specific factors involving the antagonism between ABA and GA are still not identified in the regulation of tuber formation, although many important common factors in the phytohormone signaling are associated with the process (Figure 2). In potato, *StMYB*s and *StCDPK1* are induced by both GA and ABA and their expression is regulated during potato tuber development [50,86]. The protein phosphatase type 2A (PP2A) catalytic subunits downregulated by GA can positively affect tuber formation in potato [87,88]. Meanwhile, the new ABA PYLs-PP2A signaling modulating root architecture was identified recently, in which ABA binds to PYLs and thus inhibits PP2A activity [89]. The signaling components collectively responsive to both GA and ABA offer a possible entry point into the antagonistic regulation of ABA and GA in tuber formation.

Despite the important antagonistic roles of ABA and GA in the development of stem/root tubers, tubers cannot be formed without the contributions of multiple phytohormones. Auxin is an essential hormone in all the developmental events involving the formation and maintenance of meristem [90]. Before tuber initiation, the auxin content remains relatively low [90]. However, along with stolon elongation, the auxin content increases, while GA goes down. When tubers start swelling, the content level of auxin keeps increasing and then slowly decreases after reaching a peak [90]. Cytokinin is another important phytohormone during plant development. Cytokinin and auxin play either a synergistical or an antagonistic role in meristem biology [91]. Although cytokinin is upregulated during tuberous root development, the auxin/cytokinin ratio may be the key factor for tuberization [92]. Recently, the positive role of another phytohormone, jasmonic acid, was further identified in potato with the investigation of a suppressor of JA signaling [93]. JA is increased in stolon in the initial stage of tuber formation, during which, it exerts a negative role as the inhibitor of GA synthesis [94]. In addition, there is a spectrum of other phytohormones involved in tuber formation. Hormone profiling during tuberization has been performed in various tuber crops, such as potato [94,95,96], cassava [92], yam [24,97], *R. glutinosa* [33], and sweet potato [70].

**Table 1:** The GA-and ABA-related gene expression patterns and GA and ABA profiles during stem/root tuber development in different tuber and root crops described in the article.

Species	Storage Organ	GA-and ABA-Related Gene Expression	GA and ABA Profile	Ref.
Potato (*Solanum tuberosum*)	Tuber (developing from the stolon)	*GA20ox1*: Expressed mainly in leaves. *GA2ox1*: Upregulated in the subapical zone of the stolon and growing tuber before tuber expansion. *CDPK*: Mainly located in the plasma membrane of swelling stolons and sprouting tubers. *GA3ox2*: Increased in the aerial parts and repressed in the stolons under the short-day condition.	GA_3_: The content in the tuber dropped by around 30%.	[18,29,30,31,32,33,34,41,42,43,44,45,49,50,86,98]
			ABA: Around 30% increase detected in the tuber.	[18,41,61,78,86,87,88]
Chinese arrowhead *Sagittaria trifolia*	Corm (developing from the tips of the stolon)	*KS* and *KO*: Enhanced during stolon elongation and decreased when the corm swells. *CDPK*: Upregulated.		[28]
		*ABA 8-hydroxylase*: Decreased at the initial swelling stage.		[28]
Lotus (*Nelumbo nucifera*)	Rhizome (developing from the stem)	*GAI*: Downregulated during rhizome development.		[42]
		*ABF*s, *PP2C*, *PYL*, and *SnRK2*: Different expression patterns, with some upregulated, some downregulated, and two *PP2C*s expressed high in the middle stage.		[42]
Yams (*Dioscorea opposita*)	Underground tuber (developing from the hypocotyl); and aerial tubers (bulbils)	*GA20ox1*, *GA3ox1*, and four *GA2ox*s: Significantly abundant in the early expansion stage and gradually declined along with tuber growth. Three *GID*s and three *DELLA*s: Different expression patterns in the early expansion stage and gradually declined along with tuber growth.	GA_3_ and GA_4_: Reached a peak of around 150 ng/g at 90 days after field planting and then decreased.	[23,97]
		*NCED, ZEP*, and *ABF*: Increased at the final stage. *PP2C*s: Upregulated in the early microtuber formation stage.	ABA (during tuber development): Reached a maximum of over 600 ng/g at 90 days after field planting and then decreased. ABA (in bulbil tissues): Continuously increased by around 22-fold.	[24,73,97]
Turnip (*Brassica rapa* var. *rapa*)	Taproot (developing from the hypocotyl and a part of the root)	*GA20ox*, *GA3ox*, and *GA13ox*: Increased in the early growth stage but declined during the late developmental stage. *KS*: Decreased. *GA2ox*s (except for *GA2ox8-4*): Downregulated.	GA: Most abundant active GA is GA3, and all the active GAs decreased by more than 50%.	[35]
Sugar beet (*Beta vulgaris* L.)	Taproot	*GA20-ox* and *GA3-ox*: Preferentially expressed during late development. *GA2-ox*: Preferentially expressed during early development.	GA/ABA ratio: Decreased from 1358.5 to 18.8.	[54,55,56]
		ZEP: Preferentially expressed during late development.		[55,56]
Carrot (*Daucus carota* L.)	Taproot	*GA20ox*s and *GA3ox*s: Different expression patterns, with some upregulated in the middle stage and then downregulated and some downregulated from the early development stage. *GA2ox*, *KO*, and *KS*: Upregulated and then downregulated. *KAO*: Downregulated and then upregulated.		[36,37,38]
		*NCED* and *AAO*: Upregulated in the final growth stage. *ABAH*, *PYL* and *SnRK2*: Decreased. *PP2c*: Upregulated in the early stage and then downregulated.		[36]
Radish (*Raphanus sativus* L.)	Taproot	PLYs and PP2Cs: Upregulated.		[74]
San qi (*Panax notoginseng*)	Taproot	*GAI* and *GID*: Downregulated and then upregulated.		[80]
		*PYL*: Downregulated and then upregulated. *PP2C*s: Different expression patterns, with some downregulated and then upregulated and some upregulated at the final stage. Most of *SnRK2*: Upregulated.		[80]
Sweet potato (*Ipomoea batatas*)	Tuberous root (developing from the adventitious root)	*GA3ox*: Downregulated.	GAs: Around 2.5-fold decrease in the storage root.	[51,52,70]
		*AAO*: Enhanced in the early stage and then sharply decreased until the final stage.	ABA: Increased to 6.5 nmol/g in the early stage and finally decreased to around 3 nmol/g.	[70,81]
Cassava (*Manihot esculenta*)	Tuberous root	*KS*: Mainly detected in the cortex and parenchyma of fibrous root and significantly downregulated. *CDPK1*: Upregulated during the initial stage and gradually downregulated.	ABA: Decreased in fibrous roots compared with the pretuberous roots.	[27,92]
Chinese foxglove *Rehmannia glutinosa*	Tuberous root	*GA20ox* and *GA 3-beta-dioxygenase*: Downregulated. Most of GA 2-beta-dioxygenase: Upregulated and then downregulated.	GA: Decreased by approximately 30%.	[33,75]
		*ZEP*, *AAO*, *PYL*, and most of the *NCED*s: Upregulated. Most of *ABA 8′-hydroxylase 3*: Downregulated.	ABA: Increased 2-fold.	[33,75]
Tai zi shen *Pseudostellaria heterophylla*	Tuberous root	*ZEP*: Upregulated.		[69]

Root and tuber crops are a great source of nutrition for humans. Improvement in the nutritional or mechanical properties of tuber crops requires a clear understanding of tuber organogenesis. The regulation of tuber development is influenced by multiple environmental cues depending on the regulation of hormone signaling crosstalk. The antagonism between ABA and GA can be considered as a part of the hormone signaling crosstalk. Therefore, the antagonistic role of ABA and GA during tuber development needs to be further dissected. As the model plant *A. thaliana* does not have stem/root tubers, potato becomes an important material for investigating the mechanism of tuber organogenesis. However, tuber crops include a multitude of species that may achieve tuber organogenesis through different mechanisms. Thereby, studies on the tuber organogenesis of tuber crops will be a big challenge.

## Data Availability

Not applicable.

## Abbreviations

AAO	Aldehyde oxidase
ABA	Abscisic acid
ABEBs/ABFs	ABA-responsive element binding proteins/factors
ABI5	ABA INSENSITIVE 5
bZIP	Basic leucine zipper domain
CDPKs	Ca^2+^-dependent protein kinases
GA	Gibberellin
GGPP	*trans*-Geranylgeranyl diphosphate
GID1	Gibberellin-insensitive dwarf 1
KAO	*ent*-Kaurenoic acid oxidase
KO	*ent*-Kaurene oxidase
KS	*ent*-Kaurene synthase
NCEDs	9′-cis-Epoxycarotenoid dioxygenases
ODD	Oxoglutarate-dependent dioxygenase
PP2C	Clade A protein phosphatases of type 2C
PYL	Pyrabactin resistance-like protein
SLY1	F-box proteins SLEEPY1
SnRK2	Sucrose non-fermenting-1 (SNF1)-related protein kinase 2
ZEP	Zeaxanthin epoxidase
